# Untargeted metabolomic profiling of hydrosalpinx fluid: a descriptive analysis with pelvic fluid as external reference

**DOI:** 10.3389/fendo.2026.1847941

**Published:** 2026-07-03

**Authors:** Haofei Shen, Wankun Hao, Jianxiu Zheng, Xiaorong Luo, Chunjie Zhang, Ahui Liu, Xiaoling Ma, Yuan Ding

**Affiliations:** 1 The First Hospital of Lanzhou University, Lanzhou, China; 2 The First Clinical Medical College of Lanzhou University, Lanzhou, China; 3 Gansu Maternal and Child Health Hospital, Lanzhou, China; 4 Xi’an People’s Hospital, Xi’an Fourth Hospital, Xian, China

**Keywords:** amino acid metabolism, candidate metabolites, hydrosalpinx, infertility, lipid metabolism, metabolomics

## Abstract

**Introduction:**

Hydrosalpinx is one of the common organic causes of infertility, and the accumulation of fluid in this condition can severely affect embryo implantation and development.

**Material and methods:**

We collected fallopian tube fluid from 10 patients with hydrosalpinx and normal pelvic fluid from 10 infertile women undergoing tubal patency testing as an external reference for untargeted metabolomics analysis. Metabolites were annotated using the KEGG and HMDB databases, and key metabolic disorder pathways were identified. Weighted gene co-expression network analysis (WGCNA) was then employed to screen for hub metabolites closely associated with disease status.

**Results:**

The results showed that a total of 660 significantly different metabolites were identified, of which 209 were upregulated and 451 were downregulated. Enrichment analysis indicated that these metabolites were mainly involved in the Biosynthesis of unsaturated fatty acids, Efferocytosis, Arachidonic acid metabolism, Tryptophan metabolism, etc. WGCNA further identified several core metabolites, including 5-[1-Carboxy-2-(Trimethylazaniumyl) Ethoxy]-5-Oxopentanoate, D-Dopachrome, etc.

**Conclusions:**

Compared with pelvic fluid, the metabolic profile of hydrosalpinx shows differences in lipid and amino acid metabolism, including differences in unsaturated fatty acid Biosynthesis, apoptotic cell clearance, arachidonic acid metabolic pathways, and tryptophan metabolism.

## 1 Introduction

Tubal factors account for approximately 25% of infertility cases, with hydrosalpinx representing its most severe manifestation (1). Despite the application of *in vitro* fertilization-embryo transfer (IVF-ET) in infertile patients, hydrosalpinx continues to reduce embryo implantation and pregnancy rates following assisted reproductive technology, while also increasing miscarriage rates (2, 3). Numerous theories exist regarding the reduced outcomes of assisted reproductive technologies in cases of hydrosalpinx, yet the precise causes and mechanisms remain unknown. Currently, the decreased pregnancy rate associated with hydrosalpinx is attributed to impaired embryo implantation, embryo toxicity, mechanical flushing, and alterations in the intrauterine environment. Multiple studies indicate that the fluid in the fallopian tubes is filled with tissue debris, lymphocytes, microorganisms, and cytokines, all of which may exert adverse effects on the endometrium and embryos (4). Although the cause remains unknown, abnormal protein expression levels involved in embryo implantation are observed in hydrosalpinx. Studies indicate that among women undergoing IVF with hydrosalpinx, regardless of whether the hydrosalpinx is unilateral or bilateral, the prevalence of endometritis is significantly elevated (4). Concurrently, in patients with hydrosalpinx, abnormalities in the expression of HOXA10, IGFBP1, avβ3, and their downstream genes—all of which are associated with endometrial receptivity—were identified. This may further impair embryo implantation (5). However, the precise mechanism by which hydrosalpinx alters embryo implantation rates in assisted reproductive technologies remains incompletely understood.

For the treatment of hydrosalpinx, the National Institute for Health and Care Excellence (NICE) recommends laparoscopic salpingectomy before initiating IVF (6). Meta-analysis results indicate that laparoscopic tubal resection or tubal obstruction before IVF improves pregnancy outcomes (7). Although damage can gradually repair itself after hydrosalpinx resection, recovery is a time-consuming process. In recent years, in addition to tubal resection, interventional embolization, laparoscopic tubal ligation, and tubal ostomy have been applied clinically (8, 9).

Metabolomics provides a comprehensive approach for characterizing metabolic changes under various physiological or pathological conditions. Currently, as a powerful analytical tool, metabolomics is widely applied to diverse samples, including blood, urine, follicular fluid, and endometrial fluid (10, 11). Given the practical and ethical challenges of obtaining tubal fluid from healthy fallopian tubes, pelvic fluid from infertile women without hydrosalpinx was included as an available external reference. Therefore, this study employed an untargeted metabolomics approach using ultra-high-performance liquid chromatography-tandem mass spectrometry to characterize the metabolomic profile of hydrosalpinx fluid.

## 2 Method

### 2.1 Participant sample

This study was approved by the Clinical Research and Ethics Committee of the First Affiliated Hospital of Lanzhou University (Ethics Approval Number: LDYYSZLL2025-17). All patients provided informed consent by signing the informed consent form.

This study recruited 20 women who underwent laparoscopic surgery at the Reproductive Center of Lanzhou University First Hospital between January and December 2024. The study included 10 women with hydrosalpinx and 10 women undergoing laparoscopic tubal ligation. In the hydrosalpinx group, tubal resection or proximal tubal occlusion surgery was scheduled before IVF whenever hydrosalpinx (unilateral or bilateral) was diagnosed. For the study group, inclusion criteria were as follows: 1) Laparoscopically diagnosed unilateral or bilateral hydrosalpinx (HSF), diagnosed as HS during hysterosalpingography due to ampullary dilatation without contrast agent efflux from the fimbrial end. Bilateral or unilateral HSF samples were collected from each patient. When a patient had bilateral hydrosalpinx, fluid from both tubes was pooled into a single sample to ensure that each individual contributed only one data point; thus, all samples analyzed in this study were independent. Samples were stored at -20 °C and were discarded if necessary. 2) Female infertility caused by HSF. Control group samples were obtained from women with normal pelvic fluid following laparoscopic tubal flushing. Exclusion criteria were as follows: (1) Mental or intellectual disability impairing capacity for consent; (2) Chromosomal abnormalities in either partner; (3) Uterine malformations; (4) Diagnosis of endometriosis.

The statistical analyses were performed using SPSS 22.0. Continuous variables were presented as means ± standard deviations (SD), and a t-test was used for comparisons. If normality assumptions were not met, the Mann-Whitney U test was used for comparisons. A P-value < 0.05 was considered statistically significant.

### 2.2 Sample preparation

Aliquot 100 μL of the sample into a 1.5 mL EP tube. Add 400 μL of pre-chilled extraction solvent (a 1:1 mixture of methanol and acetonitrile by volume). Mix thoroughly immediately using a vortex mixer. Perform ultrasonic treatment at low temperature to complete extraction. Next, allow the mixed sample to stand at -20 °C for 30 minutes, then centrifuge at 13,000 rpm for 15 minutes at 4 °C. Collect the supernatant from the centrifuge and thoroughly dry it using a stream of nitrogen gas. Resuspend the dried precipitate in 100 µL of resuspension solvent (prepared with a 1:1 volume ratio of acetonitrile and water). Then, assist dissolution by ultrasonication for 5 minutes at 5 °C and 40 kHz to enhance solubility. Subsequently, centrifuge the solution again at 13,000 rpm for 10 minutes under low-temperature conditions. Transfer the final supernatant to a liquid chromatography injection vial with an insert tube for subsequent LC-MS/MS analysis.

### 2.3 Preparation of QC samples

Equal volumes of all sample metabolites are pooled to prepare quality control (QC) samples. During instrumental analysis, one QC sample is inserted every 5–15 samples to monitor instrument stability and evaluate data quality.

### 2.4 Chromatography-mass spectrometry analysis

Separation was performed using an Acquity UPLC HSS T3 column (2.1 × 100 mm, 1.8 μm) with a three μL injection volume. Mobile phase A consisted of water and acetonitrile (95:5, v/v) supplemented with 0.1% formic acid. Mobile phase B was a mixture of acetonitrile, isopropanol, and water in a 47.5:47.5:5 (v/v/v) ratio, supplemented with 0.1% formic acid. The flow rate was controlled at 0.40 mL/min, and the column temperature was maintained at 40 °C. The gradient elution program in positive ion mode was as follows: During the initial phase (0–3 min), mobile phase B increased from 0% to 20%; Subsequently, over 3–4.5 min, the B phase proportion rose from 20% to 35%; Rapidly increased to 100% between 4.5–5 min and held until 6.3 min; Rapidly decreased back to 0% between 6.3–6.4 min and equilibrated until 8 min. The negative ion mode gradient was set as follows: Phase B increased from 0% to 5% between 0–1.5 min; increased to 10% between 1.5–2 min; gradually increased to 30% between 2–4.5 min; Rapidly increase to 100% within 4.5–5 min and maintain until 6.3 min; then rapidly recover to 0% within 6.3–6.4 min and continue until 8 min. Mass spectrometry analysis utilizes Q Exactive series instruments to collect data in both positive and negative ion modes, with the scan mass range set to m/z 70–1050. Key ion source parameters included: a spray voltage of 3500 V in positive mode and -3500 V in negative mode; sheath gas and auxiliary gas flow rates were set at 50 psi and 13 psi, respectively; and the auxiliary gas heating temperature was 425 °C. Fragmentation was achieved using stepwise collision energy (20–40–60 V), with data acquisition performed in data-dependent acquisition (DDA) mode.

### 2.5 Data processing

Data analysis was performed on the MajorBio cloud platform (https://cloud.majorbio.com). After the computer operation was completed, the raw data were subjected to peak detection, extraction, alignment, and integration using Progenesis QI (Waters Corporation, Milford, USA), and compound annotation was performed simultaneously using secondary mass spectrometry data (HMDB database (http://www.hmdb.ca/), Metlin (https://metlin.scripps.edu/), and Major ‘s own database). The ropls package (version 1.6.2) in R was used to perform unsupervised principal component analysis (PCA) and supervised orthogonal partial least squares discriminant analysis (OPLS-DA) to evaluate overall metabolic differences between groups. Differential metabolites were screened based on the following criteria: variable importance in the projection (VIP) in the OPLS-DA model > 1, Benjamini-Hochberg FDR < 0.05, and fold change > 1.2. Subsequently, metabolic pathway enrichment analysis was performed on the identified differential metabolites using the Kyoto Encyclopedia of Genes and Genomes (KEGG) and the Human Metabolome Database (HMDB). Additionally, weighted gene co-expression network analysis (WGCNA) was applied to construct a metabolite co-expression network, clustering metabolites with similar expression patterns into distinct modules. This approach further identified key functional modules and core differentially expressed metabolites.

## 3 Results

### 3.1 Clinical characteristics of the two patient groups

There were no significant differences between the hydrosalpinx group and the control group in terms of age, BMI, duration of infertility, baseline serum hormone levels (FSH, LH, E2, P), or AMH. (**Table 1**).

**Table 1:** Comparison of clinical features of hydrosalpinx and pelviceffusion.

Variables	Hydrosalpinx	Pelviceffusion	*p*-value
Total NO.of patients	10	10	
Ages(year)	32.00±3.74	30.30±4.50	0.37
Body mass index(kg/m^2^)	23.17±3.74	21.75±2.56	0.335
Years of infertility	3.90±2.47	2.80±1.87	0.277
Age of menarche	13.40±1.07	13.1±1.20	0.563
Menstrual cycle	29.2±2.53	29±2.62	0.864
FSH	6.86±0.66	7.06±1.22	0.653
LH	3.85±1.71	5.75±2.38	0.055
E2	33.24±8.52	39.62±12.05	0.188
AMH	3.53±2.74	2.81±1.52	0.48
Number of retrieved oocytes	16.6±6.72	13.1±7.43	0.284

FSH, follicle-stimulating hormone; LH, Luteinizing hormone; E2, 17- estradiol; AMH, Anti-müllerian hormone.

### 3.2 Multivariate analysis of metabolites

**Figure 1:**
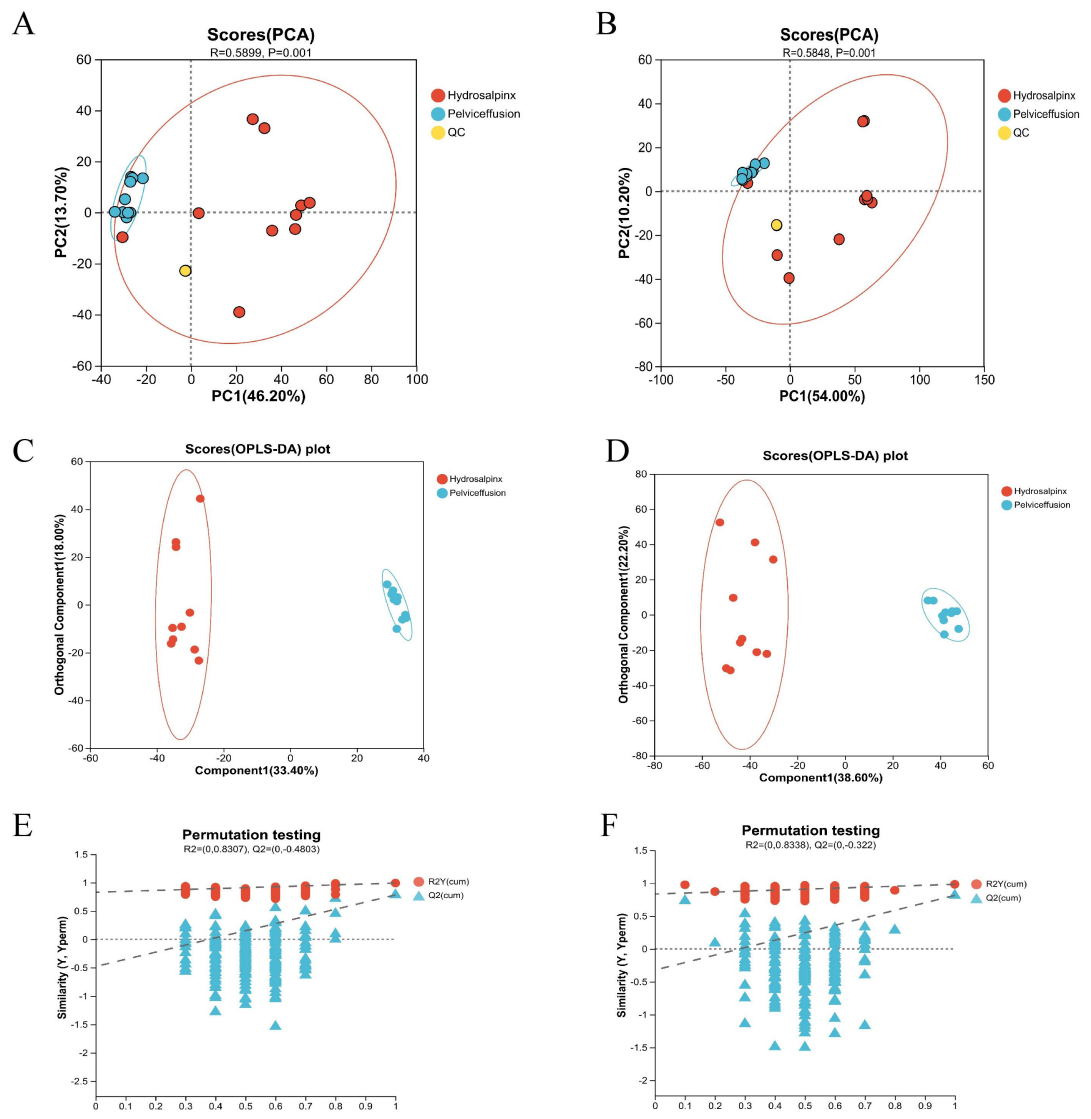
Multivariate analysis of metabolites in two groups. **(A, B)** Principal component analysis (PCA) score plots in positive **(A)** and negative **(B)** ion modes. Ellipses represent 95% confidence intervals. The tight clustering of QC samples indicates stable instrument performance. **(C, D)** Orthogonal partial least squares discriminant analysis (OPLS-DA) score plots in positive **(C)** and negative **(D)** ion modes. Red circles: HSF; blue squares: PEF. **(E, F)** OPLS-DA permutation tests in positive **(E)** and negative **(F)** ion modes. The original R²Y and Q² values are indicated by the vertical lines (solid: R²Y; dashed: Q²). Negative Q² values suggest a lack of predictive ability, which is expected given the small sample size. Abbreviations: HSF, hydrosalpinx fluid; PEF, pelvic effusion fluid; QC, quality control; PCA, principal component analysis; OPLS-DA, orthogonal partial least squares discriminant analysis.

Non-targeted metabolomics analysis was performed on both sample groups in both positive and negative ion modes, yielding a total of 18,770 mass peaks and identifying 2,882 metabolites. To investigate overall differences in metabolic profiles between groups, PCA and OPLS-DA analyses were conducted on data from both ion modes. PCA results showed that samples from the hydrosalpinx and pelvic effusion groups were distributed in different regions, but were not completely separated, suggesting some individual variation. Meanwhile, quality control (QC) samples clustered tightly (**Figures 1A, B**), indicating good instrument stability and high reproducibility. OPLS-DA effectively enhanced intergroup separation by filtering out classification-irrelevant variation through orthogonal signal correction. Scoring plots revealed a distinct separation trend, with hydrosalpinx samples clustering on the left and pelvic effusion samples distributed on the right (**Figures 1C, D**), confirming significant metabolic differences between the groups. To further assess model quality, permutation tests were conducted. In the positive ion mode, R²Y = 0.8307 and Q² = -0.4803; in the negative ion mode, R²Y = 0.8338 and Q² = -0.322 (**Figures 1E, F**). The R²Y values approached 1 in both modes, indicating high model goodness of fit. However, there is a certain risk of overfitting.

### 3.3 Screening of differentially expressed metabolites and functional enrichment analysis

**Figure 2:**
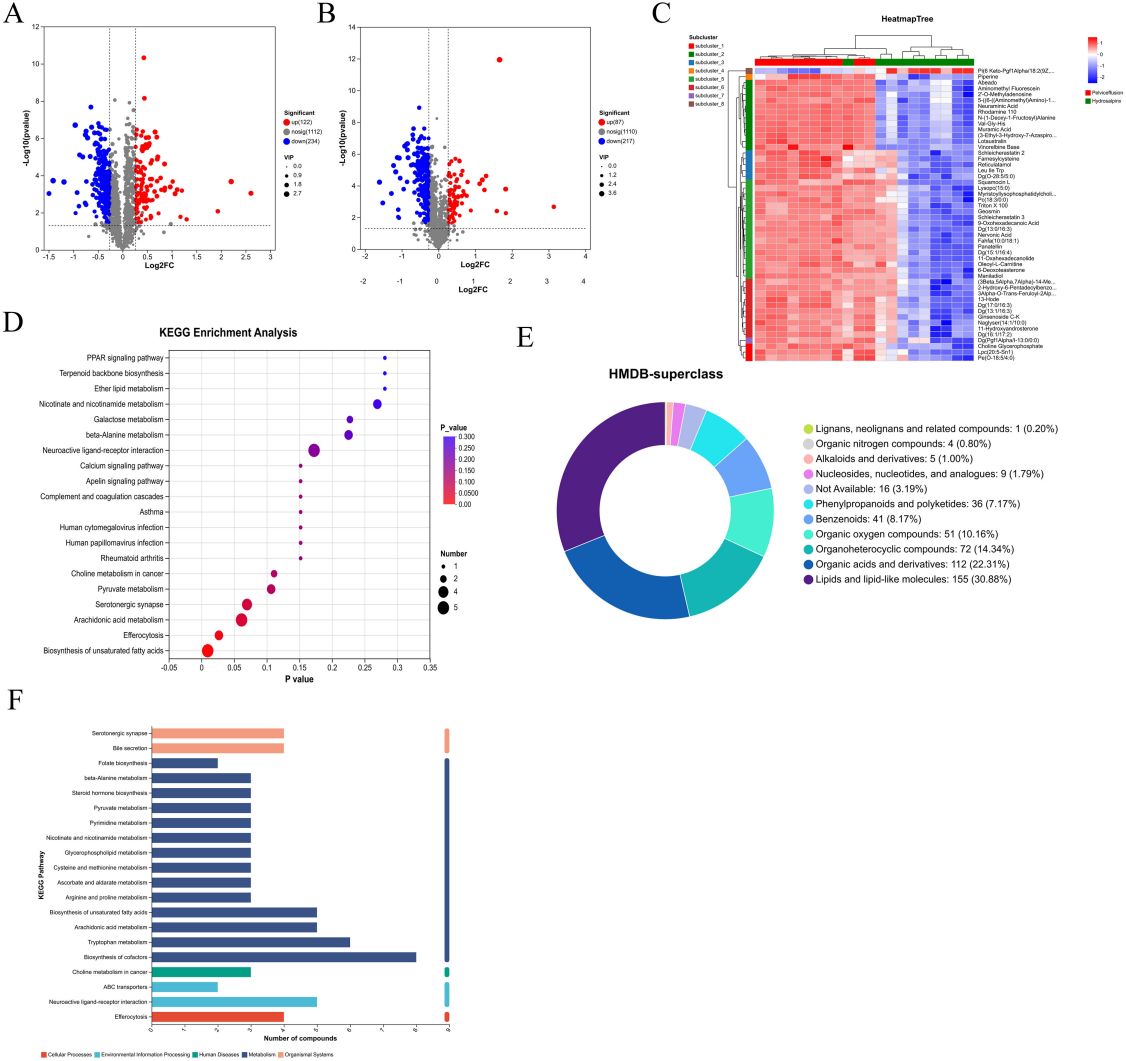
Screening of differential metabolites and functional enrichment analysis. **(A, B)** Volcano plots showing differential metabolites in positive **(A)** and negative **(B)** ion modes. Red: upregulated in hydrosalpinx fluid; blue: downregulated. A total of 660 differential metabolites (209 upregulated, 451 downregulated) were identified (FDR < 0.05, VIP > 1). **(C)** Hierarchical clustering heatmap of the top 50 differential metabolites. **(D)** KEGG pathway enrichment analysis. Significantly enriched pathways include biosynthesis of unsaturated fatty acids, efferocytosis, and arachidonic acid metabolism. **(E)** HMDB. The largest categories are lipids and lipid−like molecules, organic acids and derivatives, and organoheterocyclic compounds. **(F)** KEGG functional pathway classification (bar plot). Affected pathways include biosynthesis of cofactors, tryptophan metabolism, biosynthesis of unsaturated fatty acids, efferocytosis, and arachidonic acid metabolism.

**Figures 2A, B** display volcano plots of differentially expressed metabolites in positive and negative ion modes, respectively. Red indicates metabolites upregulated in the hydrosalpinx group, while blue denotes downregulated metabolites. A total of 660 significantly differentially expressed metabolites were identified, comprising 209 upregulated and 451 downregulated metabolites. Hierarchical clustering analysis was performed on the top 50 metabolites with the most pronounced expression differences, as shown in **Figure 2C**, visually illustrating the expression pattern disparities between the two groups.

To investigate the biological functions and categories of differentially expressed metabolites, annotation and enrichment analysis were performed using the KEGG and HMDB databases. KEGG pathway enrichment analysis revealed significant enrichment of differential metabolites in pathways such as Biosynthesis of unsaturated fatty acids, Efferocytosis, and Arachidonic acid metabolism (**Figure 2D**), suggesting that these metabolic pathways may play crucial roles in the occurrence and development of hydrosalpinx. Additionally, the HMDB primary classification annotation revealed that the differential metabolites predominantly belonged to major categories, including Lipids and lipid-like molecules, Organic acids and derivatives, and Organoheterocyclic compounds (**Figure 2E**). The KEGG pathway analysis of these differential metabolites shows that they are mainly associated with Biosynthesis of cofactors, Tryptophan metabolism, Biosynthesis of unsaturated fatty acids, Efferocytosis, and Arachidonic acid metabolism (**Figure 2F**). These findings provide data to deepen understanding of the molecular mechanisms underlying hydrosalpinx and to identify potential therapeutic targets.

### 3.4 Identification of key differentially expressed metabolites and expression patterns

**Figure 3:**
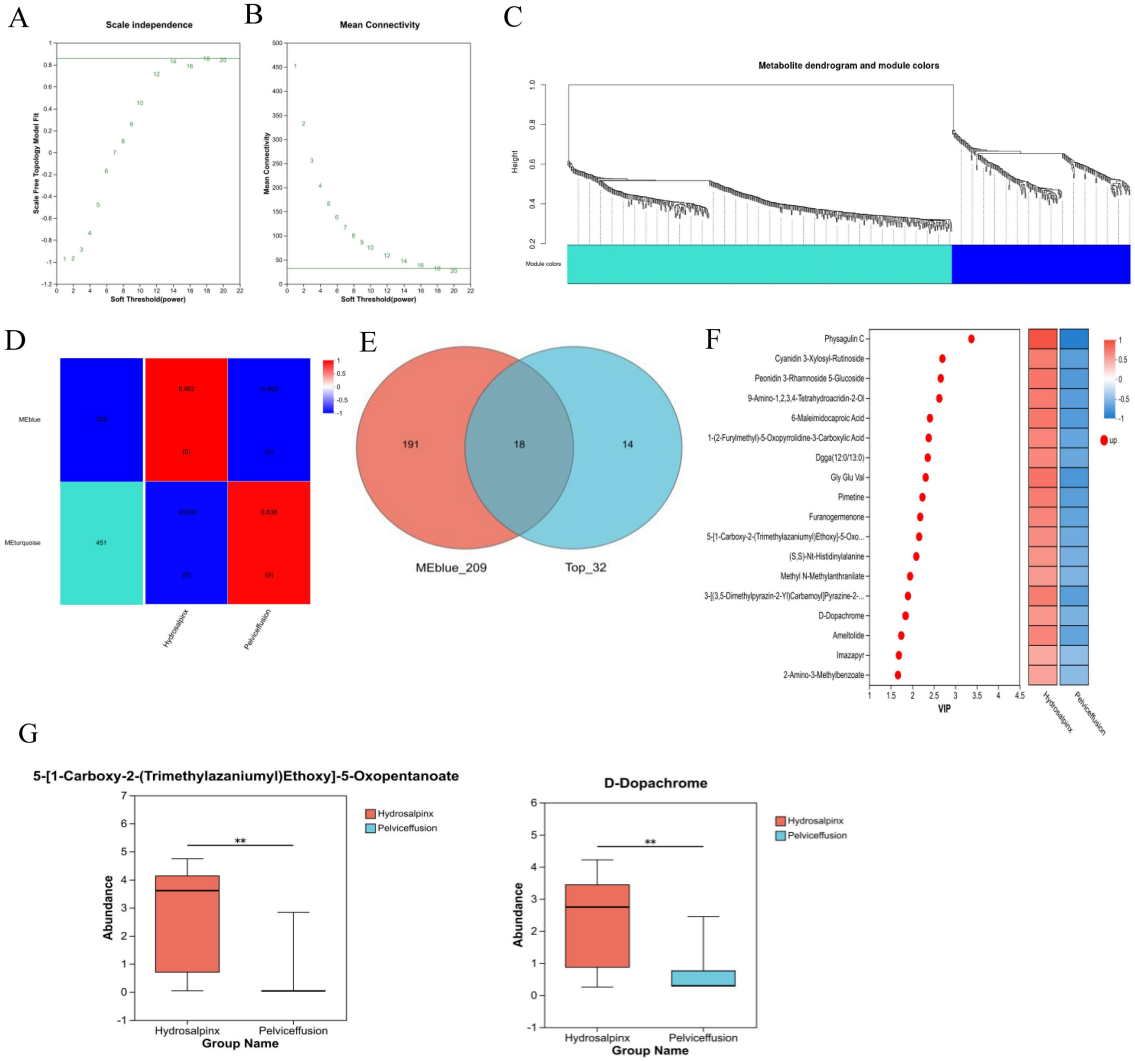
Identification of key differential metabolites. **(A, B)** Scale-free topology model fit **(A)** and mean connectivity **(B)** for soft-thresholding power selection. The optimal soft threshold was 18. **(C)** Hierarchical clustering dendrogram of metabolites. Colored blocks indicate co-expression modules. **(D)**Module-trait correlation heatmap. The MEblue module showed the strongest positive correlation with hydrosalpinx phenotype (R = 0.862, P < 0.001). **(E)**Venn diagram showing the overlap between the 209 metabolites in the MEblue module and the top 32 differential metabolites; 18 overlapping metabolites were identified as key candidates (e.g., 5-[1-Carboxy-2-(trimethylazaniumyl)ethoxy]-5-oxopentanoate, D-Dopachrome). **(F)** VIP bar chart and expression heatmap of the 18 candidate metabolites (left panel) and boxplots showing that these metabolites were significantly upregulated in hydrosalpinx fluid (right panel). WGCNA, weighted gene co-expression network analysis; VIP, variable importance in projection.

To identify key functional modules and core differentially expressed metabolites, this study employed WGCNA to construct a metabolic co-expression network. Based on scale-independence and average connectivity assessments, the optimal soft threshold was determined to be 18, at which the network exhibited robust average connectivity (**Figures 3A, B**). Using this threshold, a co-expression matrix was constructed and subjected to hierarchical clustering analysis (**Figure 3C**). After merging highly similar modules, two distinct metabolic clustering modules were obtained. Among these, the MEblue module showed the strongest correlation with the hydrosalpinx/pelvic effusion phenotype (R = 0.862, P < 0.001; **Figure 3D**), suggesting that its 209 metabolites may be closely associated with the disease state. Further intersecting all metabolites in the MEblue module with the top 32 most significant differential metabolites identified eighteen key differential metabolites (**Figure 3E**): 5-[1-Carboxy-2-(Trimethylazaniumyl) Ethoxy]-5-Oxopentanoate, D-Dopachrome, etc. The VIP bar chart visually illustrates the variable importance and expression trends of these nine metabolites between the two groups (**Figure 3F**). Furthermore, as shown in **Figure 3G**, these metabolites were significantly upregulated in the hydrosalpinx group, suggesting their potential role in the disease.

## 4 Discussion

This study conducted untargeted metabolomic sequencing of hydrosalpinx fluid and used peritoneal fluid from infertile women without hydrosalpinx as a control. However, given that these two fluids originate from different anatomical locations, we regard the observed differences as preliminary metabolic characteristics of hydrosalpinx fluid, rather than validated disease-specific biomarkers.

We identified 209 upregulated and 451 downregulated metabolites. KEGG pathway enrichment analysis showed that the differential metabolites were significantly enriched in the Biosynthesis of unsaturated fatty acids and efferocytosis pathways. In addition, although not reaching statistical significance, a considerable number of altered metabolites were involved in arachidonic acid metabolism and tryptophan metabolism, suggesting possible perturbations in these pathways. Dysregulation of the significantly enriched pathways may reflect the local metabolic environment of hydrosalpinx and could potentially influence reproductive outcomes by modulating lipid metabolism, inflammatory balance, and apoptotic cell clearance. Below, we discuss in detail the potential mechanisms underlying these differential metabolites.

Our KEGG pathway enrichment analysis identified the Biosynthesis of unsaturated fatty acids as significantly perturbed in hydrosalpinx fluid, with five representative very-long-chain and polyunsaturated fatty acids—nervonic acid, 13,16−docosadienoic acid, erucic acid, eicosadienoic acid, and eicosapentaenoic acid (EPA)—being consistently downregulated compared with pelvic fluid. These five metabolites represent only a fraction of the broader pathway; however, their coordinated downregulation, together with the significant pathway-level enrichment, strongly suggests a systemic alteration in unsaturated fatty acid metabolism within the hydrosalpinx environment.

Notably, two of these fatty acids have been shown to be relevant to female fertility and ovarian function. Nervonic acid is a well-known component of metabolic syndrome: recent work demonstrated that nervonic acid triggers ovarian inflammation by inducing mitochondrial oxidative stress and activating the NLRP3/IL-1β pathway (12), thereby strongly influencing ovarian function and embryo survival. Eicosapentaenoic acid (EPA) is a well-known anti−inflammatory fatty acid and a direct precursor of E−series resolvins, specialized pro−resolving mediators (SPMs) that actively terminate inflammation (13–15); it also attenuates inflammatory cytokine production and reduces macrophage infiltration in the reproductive tract (16). Thus, the reduction of EPA in hydrosalpinx fluid raises the possibility that the local capacity for inflammatory resolution may be impaired, potentially facilitating the persistent inflammatory state characteristic of hydrosalpinx.

From a reproductive perspective, unsaturated fatty acids, particularly polyunsaturated fatty acids, exert profound effects on endometrial receptivity, embryo development, and implantation. They modulate membrane fluidity and lipid raft organization, serve as essential substrates for prostaglandin synthesis, and influence steroid hormone production and cell signaling pathways crucial for decidualization and trophoblast invasion (17). Indeed, prostaglandins derived from arachidonic acid are indispensable for embryo implantation, regulating vascular permeability, stromal decidualization, and trophoblast invasion (18). A recent meta−analysis has further confirmed that omega−3 intake significantly improves pregnancy and fertilization rates in women.

In summary, we found that the Biosynthesis of unsaturated fatty acids was significantly altered in hydrosalpinx fluid, with five specific fatty acids consistently lower than in normal pelvic fluid. These results indicate a disturbance in lipid metabolism within the hydrosalpinx environment. Our data do not prove that these fatty acid changes directly cause lower implantation or pregnancy rates. However, they add to the known list of abnormal components in hydrosalpinx fluid. Previous studies have shown that abnormal fatty acid metabolism can affect reproduction and that hydrosalpinx fluid is toxic to embryos. Therefore, the fatty acid alterations we observed should be further investigated as possible contributors to this toxicity. Future experiments, such as *in vitro* embryo culture with controlled lipid supplementation, are needed to determine whether restoring normal fatty acid levels can reduce the harmful effects of hydrosalpinx fluid.

KEGG pathway enrichment analysis also identified efferocytosis as significantly perturbed in hydrosalpinx fluid. Efferocytosis is the process by which phagocytes—primarily macrophages—clear apoptotic cells. This process is essential for maintaining tissue homeostasis, resolving inflammation, and preventing autoimmunity. Dysfunction of efferocytosis has been implicated in a wide range of chronic inflammatory conditions, where failure to clear apoptotic debris leads to secondary necrosis and persistent inflammation (19).

Efferocytosis is particularly important in the female reproductive tract. During the menstrual cycle, and especially during embryo implantation, the endometrium undergoes extensive apoptosis and tissue remodeling, which must be efficiently cleared to maintain a receptive, non−inflammatory environment for the implanting embryo. Recent research has shown that dysregulation of efferocytosis contributes to reproductive challenges, including low pregnancy rates, miscarriages, and implantation failures (20). Moreover, defective efferocytosis can lead to compromised implantation, recurrent miscarriages, and unsuccessful assisted reproductive procedures (20). In support of this, a 2022 study demonstrated that disruption of the IL−33/ST2−AXL−efferocytosis axis in decidual macrophages leads to pregnancy failure through metabolic reprogramming, providing direct mechanistic evidence linking efferocytosis dysfunction to adverse pregnancy outcomes (21).

The finding that efferocytosis is significantly enriched in hydrosalpinx fluid is clinically relevant because hydrosalpinx is inherently a chronic inflammatory condition. The fallopian tube in hydrosalpinx contains abundant cellular debris, inflammatory cells, and apoptotic epithelial cells, which must be cleared to prevent perpetuation of local inflammation. If efferocytosis is impaired or the local environment overwhelms this clearance mechanism, apoptotic cells may undergo secondary necrosis, releasing damage−associated molecular patterns (DAMPs) that further amplify inflammation and tissue damage. This vicious cycle could, at least in part, explain the persistent inflammatory state characteristic of hydrosalpinx and its resistance to resolution.

From a reproductive perspective, when hydrosalpinx fluid refluxes into the uterine cavity—a phenomenon known to occur—it may carry with it the same metabolic features that disrupt local efferocytosis. The resulting impairment of apoptotic cell clearance in the endometrium during the implantation window could create a pro−inflammatory microenvironment that interferes with embryo attachment and early development.

In summary, the significant enrichment of the efferocytosis pathway in hydrosalpinx fluid points to a clinically relevant metabolic alteration. While our data do not prove that this alteration directly causes the reduced implantation and pregnancy rates in hydrosalpinx patients, it adds to the increasing evidence that efferocytosis dysfunction is closely linked to reproductive failure. The metabolic features identified in this study may contribute to the hostile microenvironment created by hydrosalpinx fluid, and they warrant further investigation as potential therapeutic targets.

Among the candidate metabolites identified by WGCNA, D-Dopachrome was significantly elevated in hydrosalpinx fluid. D-Dopachrome is an intermediate in the melanin biosynthesis pathway, and its tautomerase (DDT, also known as MIF−2) is a pro−inflammatory cytokine of the macrophage migration inhibitory factor (MIF) superfamily. DDT binds to the CD74/CD44 receptor complex, activates MAPK signaling, and induces downstream inflammatory pathways, including COX2/PGE_2_. Neutralization of DDT *in vivo* has been shown to reduce inflammation (22, 23) significantly.

Our finding that D−Dopachrome is significantly elevated in hydrosalpinx fluid, therefore, suggests that the DDT−mediated inflammatory pathway may be overactive in the hydrosalpinx environment. When such fluid refluxes into the uterine cavity, it can create a pro−inflammatory milieu hostile to embryo implantation.

However, several limitations need to be acknowledged. First, the sample size of this study is relatively small (n=10 per group), which limits statistical power, reduces generalizability, and affects the stability of WGCNA-derived modules. Therefore, these differential metabolites are only considered exploratory. Consequently, the separation observed in the OPLS-DA score plot should be viewed only as descriptive, and the differential metabolites identified through VIP > 1 remain hypothesis-generating rather than validated biomarkers. Second, the metabolites detected in this study are based on level 2 mass spectrometry data, and most metabolite annotations are based on putative identification levels. Among the 18 metabolites identified in this study through WGCNA analysis, most appear to be exogenous compounds, so we remain cautious in interpreting them. Third, key potential confounding factors, including menstrual cycle stage, inflammatory or infection status, medication use, and microbiome-related effects, were not fully assessed and may influence metabolomic features. Fourth, although our discussion mentions potential effects on assisted reproductive outcomes (such as embryo implantation and pregnancy rates), this study did not collect direct clinical outcome data from enrolled patients, such as IVF success rates or implantation outcomes. Therefore, any linking of the observed metabolic changes to reproductive outcomes should be regarded purely as speculative and hypothesis-generating. Future studies with prospective follow-up of clinical endpoints are needed to establish any causal or prognostic relationships. Finally, this study lacks an independent validation cohort. Therefore, the identified candidate metabolites should be considered preliminary findings and require validation in larger, multicenter studies.

## Data Availability

The original contributions presented in the study are included in the article/supplementary material. Further inquiries can be directed to the corresponding authors.

## References

[1] Ng KYB Cheong Y . Hydrosalpinx - salpingostomy, salpingectomy or tubal occlusion. Best Pract Res Clin Obstet Gynaecol. (2019) 59:41–7. doi: 10.1016/j.bpobgyn.2019.01.011 30824209

[2] Hao HJ Wang ZH Feng L Zhao XL Chen X . Which patients with hydrosalpinx will benefit more from reproductive surgery to improve natural pregnancy outcomes?: a systematic review and meta-analysis. Med (Baltimore). (2023) 102:e32806. doi: 10.1097/md.0000000000032806 36827021 PMC11309686

[3] Harb H Al-Rshoud F Karunakaran B Gallos ID Coomarasamy A . Hydrosalpinx and pregnancy loss: a systematic review and meta-analysis. Reprod BioMedicine Online. (2019) 38:427–41. doi: 10.1016/j.rbmo.2018.12.020 30665848

[4] Bouet PE Antaki R Rio C Boileau-Savary C Boguenet M Vielle B . High prevalence of chronic endometritis in women diagnosed with hydrosalpinx before *in vitro* fertilization treatment. J Minim Invasive Gynecol. (2025) 32:793–9. doi: 10.1016/j.jmig.2025.04.016 40320204

[5] Li T Lu F Wu C Cai YL Yang L Cai H . Study of hydrosalpinx on endometrial growth and expression of HOXA10mRNA and related factors. Heliyon. (2023) 9:e17063. doi: 10.1016/j.heliyon.2023.e17063 37342578 PMC10277592

[6] Ng KYB Cheong Y . Hydrosalpinx – salpingostomy, salpingectomy or tubal occlusion. Best Pract Res Clin Obstetrics Gynaecology. (2019) 59:41–7. doi: 10.1016/j.bpobgyn.2019.01.011 30824209

[7] Melo P Georgiou EX Johnson N van Voorst SF Strandell A Mol BWJ . Surgical treatment for tubal disease in women due to undergo *in vitro* fertilisation. Cochrane Database Syst Rev. (2020) 10:Cd002125. doi: 10.1002/14651858.cd002125.pub4 33091963 PMC8094448

[8] Qi Y Zhang J Tan L . Comparative outcomes of interventional embolization and laparoscopic tubal ligation on frozen-thawed embryo transfer success in women with hydrosalpinx. Eur J Obstetrics Gynecology Reprod Biol. (2025) 309:143–9. doi: 10.1016/j.ejogrb.2025.03.046 40147403

[9] Cohen A Almog B Tulandi T . Hydrosalpinx sclerotherapy before *in vitro* fertilization: systematic review and meta-analysis. J Minimally Invasive Gynecology. (2018) 25:600–7. doi: 10.1016/j.jmig.2017.12.004 29248666

[10] Wei Y Zhang Z Zhang Y Li J Ruan X Wan Q . Nontargeted metabolomics analysis of follicular fluid in patients with endometriosis provides a new direction for the study of oocyte quality. MedComm (2020). (2023) 4:e302. doi: 10.1002/mco2.302 37265938 PMC10229744

[11] Qian Y Tong Y Zeng Y Huang J Liu K Xie Y . Integrated lipid metabolomics and proteomics analysis reveal the pathogenesis of polycystic ovary syndrome. J Transl Med. (2024) 22:364. doi: 10.21203/rs.3.rs-3185871/v1 38632610 PMC11022415

[12] Zeng X Fan X Yu H Cai S Zhou L Wu H . Nervonic acid triggered ovarian inflammation by inducing mitochondrial oxidative stress to activate NLRP3/ IL-1β pathway. J Adv Res. (2025) 73:73–91. doi: 10.1016/j.jare.2024.08.028 39181200 PMC12225940

[13] López-Vicario C Rius B Alcaraz-Quiles J García-Alonso V Lopategi A Titos E . Pro-resolving mediators produced from EPA and DHA: overview of the pathways involved and their mechanisms in metabolic syndrome and related liver diseases. Eur J Pharmacol. (2016) 785:133–43. doi: 10.1016/j.ejphar.2015.03.092 25987424

[14] Bäck M . Icosapent ethyl in cardiovascular prevention: resolution of inflammation through the eicosapentaenoic acid - resolvin E1 - ChemR23 axis. Pharmacol Ther. (2023) 247:108439. doi: 10.1016/j.pharmthera.2023.108439 37201735

[15] Serhan CN Libreros S Nshimiyimana R . E-series resolvin metabolome, biosynthesis and critical role of stereochemistry of specialized pro-resolving mediators (SPMs) in inflammation-resolution: preparing SPMs for long COVID-19, human clinical trials, and targeted precision nutrition. Semin Immunol. (2022) 59:101597. doi: 10.1016/j.smim.2022.101597 35227568 PMC8847098

[16] Yamashita A Kawana K Tomio K Taguchi A Isobe Y Iwamoto R . Increased tissue levels of omega-3 polyunsaturated fatty acids prevents pathological preterm birth. Sci Rep. (2013) 3:3113. doi: 10.1038/srep03113 24177907 PMC3814804

[17] Chen M Zheng Z Shi J Shao J . Insight on polyunsaturated fatty acids in endometrial receptivity. Biomolecules. (2021) 12(1):36. doi: 10.3390/biom12010036 35053184 PMC8773570

[18] Sugimoto Y Aikawa S Inazumi T Hirota Y . Roles of prostaglandin signaling in implantation and decidualization. Prog Lipid Res. (2025) 100:101357. doi: 10.1016/j.plipres.2025.101357 41043556

[19] Li G Xu J Tian X Xiao J Long J Chen Y . Efferocytosis: the art of cellular clearance and novel perspectives in disease therapy. Mol Cancer. (2025) 24:268. doi: 10.1186/s12943-025-02484-7 41126210 PMC12548151

[20] Bavarsad SB Shahryarhesami S Karami N Naseri N Tajbakhsh A Gheibihayat SM . Efferocytosis and infertility: implications for diagnosis and therapy. J Reprod Immunol. (2025) 167:104413. doi: 10.1016/j.jri.2024.104413 39631138

[21] Sheng YR Hu WT Shen HH Wei CY Liu YK Ma XQ . An imbalance of the IL-33/ST2-AXL-efferocytosis axis induces pregnancy loss through metabolic reprogramming of decidual macrophages. Cell Mol Life Sci. (2022) 79:173. doi: 10.1007/s00018-022-04197-2 35244789 PMC11073329

[22] Ji H Zhang Y Chen C Li H He B Yang T . D-dopachrome tautomerase activates COX2/PGE(2) pathway of astrocytes to mediate inflammation following spinal cord injury. J Neuroinflamm. (2021) 18:130. doi: 10.21203/rs.3.rs-303168/v1 34116703 PMC8196514

[23] Merk M Mitchell RA Endres S Bucala R . D-dopachrome tautomerase (D-DT or MIF-2): doubling the MIF cytokine family. Cytokine. (2012) 59:10–7. doi: 10.1016/j.cyto.2012.03.014 22507380 PMC3367028

